# Additions to the Limoniidae and Pediciidae fauna of Morocco, with an updated checklist (Diptera, Tipuloidea)

**DOI:** 10.3897/zookeys.563.7384

**Published:** 2016-02-15

**Authors:** Ouafaa Driauach, Boutaïna Belqat

**Affiliations:** 1 Laboratory «Ecology, Biodiversity and Environment», Department of Biology, Faculty of Sciences, University Abdelmalek Essaâdi, Tétouan, 93030, Morocco

**Keywords:** Morocco, new records, North Africa, Short-palped crane flies, updated checklist

## Abstract

Eighteen species of Limoniidae and two species of Pediciidae are recorded for the first time in Morocco, of which 15 species are new to North Africa. An updated checklist of Moroccan short-palped craneflies (Limoniidae and Pediciidae) is appended, containing 73 species in 25 genera.

## Introduction

Limoniidae and Pediciidae belong to the Tipuloidea together with Cylindrotomidae and Tipulidae. The Limoniidae comprise more than 741 species already known in the Westpalaeartic region, from which only 102 species are known to occur in Morocco (49), Algeria (32), Tunisia (9), Libye (1) and Egypt (11). The Pediciidae are the second smallest family of the Tipuloidea, with 77 recognized taxa in the Westpalaeartic of which only 4 were recorded from North Africa, in Morocco (Driauach et al. 2013, Oosterbroek 2015).

Adults of Limoniidae and Pediciidae are associated with moist environments. They are usually collected from damp vegetation along the borders of various water bodies and from damp forests (Starý et al. 2005, De Jong et al. 2008). Their immature stages are usually found in a wide range of aquatic and semi-aquatic habitats (Reusch and Oosterbroek 1997).

Limoniidae and Pediciidae are poorly known in Morocco. They only received sporadic faunistic records made by European entomologists (Pierre 1922a, b, Pierre 1924, Séguy 1941a, b, Vaillant 1956, Starý 1971, Starý 1974, Savchenko et al. 1992, Eiroa 2000, Pârvu et al. 2006, Starý 2006, Starý and Oosterbroek 2008, Starý 2009, Starý 2011).

Only a single attempt was made to summarize the knowledge of the Moroccan fauna in the first checklist of the short-palped crane flies of Morocco (Driauach et al. 2013) and very recent research also revealed a new species, *Baeoura staryi* from Morocco including an identification key of the genus *Baeoura* in the West Palaearctic species (Driauach and Belqat 2015).

Prior to the present study, there were 53 species recorded from Morocco (Oosterbroek 2015). New findings increase the number of Moroccan Limoniidae and Pediciidae to 73. Altogether 44 species of Limoniidae and Pediciidae are collected and recorded in this work. Of these, 18 species of Limoniidae and two species of Pediciidae are recorded for the first time in Morocco of which 15 species are new to North Africa.

## Material and methods

The material presented in this work come from field campaigns carried out by the authors between 2011 and 2015 in 77 sites distributed over a mountainous area (Rif, High Atlas, Middle Atlas, Beni Snassen) and arid area (Sahara) of Morocco. A total of 564 specimens of short-palped crane flies (515 specimens of Limoniidae and 49 specimens of Pediciidae) were identified. Adults were mostly collected by sweeping vegetation with entomological hand net whereas other specimens were reared in the laboratory from the substratum taken from several zones of water bodies margins and kept under the laboratory conditions. Fresh material was stored in 70% alcohol. For identification, the male terminalia, if necessary, were prepared by boiling in a solution of 10% KOH and preserved in glycerine. All the specimens were collected by the authors and are deposited in the Diptera Collection of the Faculty of Sciences, University Abdelmalek Essaâdi, Tétouan, Morocco. The Distribution as it is given for individual species is based on Oosterbroek (2015).

The checklist of Moroccan short-palped craneflies is updated. New records are marked with an asterisk (*) and bold set text. A brief description of the material examined is given and the distribution of the identified species is provided, together with notes on the breeding sites (Table 1).

**Table 1.** Sampling sites (in alphabetic order) for the adults of Limoniidae and Pediciidae in Moroccan regions, 2011–2015, with localities, geographical coordinates and altitudes.

Site	Locality	Province	Altitude (m)	Geographical coordinates	Type of habitat
**Rif Mts**					
Tributary of Oued El Fondak	Aïn Lahcen, Khandek Melouka	Tétouan	291	35°33.544'N, 05°34.995'W	River of the middle course
Tributary of Oued El Kebir	Route de Beni Yedder	Chefchaouen	145	35°25.864'N, 05°27.610'W	River of the middle course
Tributary of Oued Hachef	Dar Chaoui	Tanger-Assila	47	35°31.458'N, 05°43.771'W	Small stream
Tributary of Oued Majjou	Nord Majjou	Chefchaouen	786	35°06.730'N, 05°11.309'W	Small stream
Tributary of Oued Ouara	Khandek Romana, Jbel Khizana	Chefchaouen	1003	35°02.579N 05°12.872'W	River of the middle course
Tributary of Oued Taïda	Sidi Brahim Ben Arrif	Larache	860	35°20.398'N, 05°32.471W	River of the higher course
Tributary of Oued Zarka	Zarka	Chefchaouen	128	35°31.220'N, 05°20.461'W	Stream
Aïn Afersiw	Mezine	Chefchaouen	746	35°05.945'N, 05°20.439'W	Spring
Aïn Bab Tariouente	Ânasar	Chefchaouen	1440	35°01.110'N, 05°00.586'W	Spring and stream
Aïn Boughaba	Jbel Bou Bessoui, Cedar Forest	Chefchaouen	1526	34°58.779'N, 04°46.366'W	Spring
Aïn Chouk	Aïn Chouk	Larache	5	35°09.803'N, 06°06.473'W	Marsh
Aïn El Ma Bared	Bouzthate	Chefchaouen	1246	35°00.333'N, 05°12.105'W	Spring and irrigation canal
Aïn El Maounzil	Parc National de Talassemtane	Chefchaouen	1106	35°04.577'N, 05°10.406'W	Spring and brook
Aïn El Malâab	Parc National de Talassemtane	Chefchaouen	1278	35°05.509'N, 05°09.443'W	Spring and brook
Aïn Quanquben	Jbel Bou Bessoui	Chefchaouen	1600	34°57.750'N, 04°40.783'W	Spring
Aïn Mâaze	Jbel Bouhachem	Larache	1294	35°14.381'N, 05°26.316'W	Spring
Aïn Ras El Ma	Majjou	Chefchaouen	856	35°06.873'N, 5°11.388'W	Spring
Aïn Sidi Brahim Ben Arrif (Site 1)	Route moulay Abdessalam	Larache	882	35°20.415'N, 05°32.535'W	Small stream
Aïn Sidi Brahim Ben Arrif (Site 2)	Route moulay Abdessalam	Larache	897	35°20.398'N, 05°32.712'W	Spring and brook
Aïn Takhninjoute	Bab Rouida, Parc National de Talassemtane	Chefchaouen	1512	35°06.881'N, 05°08.270'W	Spring
Âounsar Aheramen	Majjou	Chefchaouen	855	35°06.162'N, 05°10.739'W	Spring
Barrage Ajras	Aïn Lahcen	Tétouan	49	35°33.833'N, 05°29.902'W	Barrage
Barrage Moulay Bouchta	Larbaa Beni Hassan	Tétouan	364	35°15.864'N, 05°21.221'W	Barrage
Cascade Chrafate	Chrafate	Chefchaouen	820	35°03.997'N, 05°06.434'W	Waterfall
Cascade Zarka	Zarka	Tétouan	147	35°31.211'N, 05°20.477'W	Waterfall
Daya Aïn Jdioui	Aïn Jdioui	Tanger-Assilah	5	35°34.074'N, 05°55.499'W	Pond
Daya Amsemlil	Jbel Bouhachem	Tétouan	1059	35°15.596'N, 05°25.917'W	Bog
Daya El Ânassar	Bab Berred	Chefchaouen	1183	35°00.788'N, 04°57.419'W	Bog
Daya Fifi	Fifi	Chefchaouen	1202	35°1.367'N, 05°12.335'W	Bog
Daya Jbel Zemzem	Jbel Zemzem	Tétouan	233	35°45.553'N, 05°22.340'W	Pond
Daya Mezine	Mezine	Chefchaouen	778	35°06.104'N, 5°21.177'W	Pond
Daya Mghara	Dar Chaoui	Tétouan	44	35°31.471'N, 05°42.963'W	Pond
Daya Mtahen	Jbel Bouhachem	Larache	966	35°16.195'N, 05°26.158'W	Pond
Daya near Aïn Afersiw	Mezine	Chefchaouen	716	35°06.069'N, 5°20.337'W	Pond
Daya Tazia	Route Moulay Abdessalam	Larache	721	35°20.814'N, 05°33.139'W	Pond
Daya Rmali	El Malâab, Parc National de Talassemtane	Chefchaouen	1276	35°05.563'N, 05°09.488'W	Pond
Daya Afrate	Tanaqoub	Chefchaouen	600	35°05.634'N, 05°26.028'W	Bog
Lac Badriouen	Badriouen	Tanger	34	35°42.083'N, 05°52.116'W	Lack
Maison forestière	Parc National de Talassemtane	Chefchaouen	1674	35°08.076'N, 05°08.262'W	Spring and brook
Marj El Kheyl	Jbel Bouhachem	Tétouan	989	35°15.985'N, 05°26.209'W	Marshland
Oued Aârate	Dardara	Chefchaouen	269	35°07.381'N, 05°17.456'W	River of the middel course
Oued Aârkoub	Aârkoub	Chefchaouen	140	35°15.943'N, 04°50.631'W	River of the middel course
Oued Abou Bnar	Douar Abou Bnar, Parc National de Talassemtane	Chefchaouen	1254	35°10.977'N, 05°08.005'W	River of the higher course
Oued Amsa	Amsa	Tétouan	13	35°31.593'N, 05°13.821'W	Downstream of the river
Oued El Kanar	Stehate	Chefchaouen	50	35°17.233'N, 04°59.639'W	Downstream of the river
Oued Amsemlil (Site 1)	Jbel Bouhachem	Larache	1059	35°15.614'N, 05°25.943'W	River of the higher course
Oued Amsemlil (Site 3)	Jbel Bouhachem	Larache	1134	35°15.635'N, 05°26.134'W	River of the higher course
Oued at 15 Km from Fifi	Bouzthate	Chefchaouen	1256	35°00.805'N, 05°12.365'W	River of the higher course
Oued Azila	Azila, Jbel Tidghine	Al Hoceïma	1601	34°52.028'N, 04°32.609'W	River of the higher course
Oued El Kebir	Route Vers Beni Yedder	Tétouan	89	35°27.351'N, 05°25.796'W	River of the middel course
Oued El Koub	Souk El Had	Chefchaouen	124	35°01.298'N, 05°25.333'W	Waterfall
Oued Farda	Akchour	Chefchaouen	394	35°14.147'N, 05°10.576'W	River of the higher course
Oued Jnane Niche	Jnane Niche	Chefchaouen	36	35°17.019'N, 04°51.233'W	River of the middel course
Oued Loukous	Souk El Had	Chefchaouen	148	35°01.317'N, 05°25.246'W	River of the middle course
Oued Kelâa	Akchour	Chefchaouen	303	35°14.268'N, 05°10.452'W	River of the higher course
Oued Madissouka.	Madissouka, Parc National de Talassemtane	Chefchaouen	1367	35°10.622'N, 05°08.400'W	River of the higher course
Oued Majjou (Hafa meqlouba)	Majjou	Chefchaouen	825	35°06.175'N, 05°10.836'W	River of the higher course
Oued Majjou (Pont)	Majjou	Chefchaouen	786	35°06.730'N, 05°11.309'W	River of the higher course
Oued Majjou (Zaouiet El habtiyne)	Zaouiet El Habtiyne	Chefchaouen	800	35°06.784'N, 05°11.784'W	River of the higher course
Oued Mlila	Route de Ouazzane	Ouazzane	172	35°02.086'N, 05°22.133'W	River of the middle course
Oued Ouara	Ikedjiouen	Chefchaouen	680	35°3.987'N, 05°14.05'W	River of the middle course
Oued Ouringa	Jebha	Chefchaouen	17	35°11.438'N, 04°41.150'W	Downstream of the river
Oued Sidi Yahya Aârab	Sidi Yahya Aârab	Chefchaouen	62	35°17.545'N, 04°53.503'W	River of the middel course
Oued Taïda	Taïda	Larache	505	35°22.250'N, 05°32.355'W	River of the middel course
Oued Tamerte	Belota	Chefchaouen	114	34°57.607'N, 05°32.061'W	River of the middel course
Oued Tizekhte	Larbaa Beni Hassan	Tétouan	728	35°18.600'N, 05°23.097'W	Stream of the middle course
Oued Tkaraâ	Jbel Bouhachem	Larache	959	35°16.063'N, 05°25.829'W	River of the higher course
Oued Zarka	Kitane	Tétouan	52	35°32.412'N, 05°20.393'W	River of the middel course
Oued Zendoula	15 km North Ouazzane	Ouazzane	108	34°55.707'N, 05°31.942'W	River of the middel course
Seguia Mtahen	Mtahen, Jbel Bouhachem	Larache	983	35°16.226'N, 05°26.212'W	Small stream
**Beni Snassen**					
Grotte du Chameau	Zegzel, Beni Snassen	Berkan	427	34°50.447'N, 02°21.532'W	Irrigation canal
Oued Tafoughalt	Tafoughalt, Beni Snassen,	Berkan	751	34°48.941'N, 02°24.471'W	River of the Middle course
Oued Beni Ouachekradi	Beni Snassen	Berkan	534	34°50.974'N, 02°16.217'W	River of the middle course
**Middel Atlas Mts**					
Barrage Allal El Fassi	Gîte Aït Ayoub	Sefrou	537	33°55.446'N, 4°40.558'W	Dam
**High Atlas Mts**					
Oued Sidi Fares	Sidi Fares, Parc National de Toubkal	Marrakech	1807	31°14.029'N, 07°52.813'W	River of the higher course
Assif Haouz	Imlil, Parc National De Toubkal	Marrakech	1896	31°07.424'N, 7°55.083'W	Stream
**Sahara**					
Oued Zag	Zag	Assa-Zag	400	28°01.344'N, 09°17.926'W	River of the middle course

## Systematic part

### Limoniidae: Chioneinae

#### Cheilotrichia (Empeda) cinerascens

Taxon classificationAnimaliaDipteraLimoniidae

(Meigen, 1804)

##### Material examined.

Oued Ouara, 1♂, 2♀♀, 23.XI.2012; Oued Amsa, 2♂♂, 13.XII.2013; Oued Majjou (Hafa meqlouba), 1♂, 10.V.2014; Grotte du Chameau, 1♂, 3♀♀, 24.XI.2014; Aïn Quanquben, 1♂, 28.IV.2015 (sweep net).

##### Distribution.

Europe, Morocco, Georgia, Turkey, Cyprus, Israel, Iran. First record from Beni Snassen.

#### Cheilotrichia (Empeda) fuscohalterata

Taxon classificationAnimaliaDipteraLimoniidae

(Strobl, 1906)

##### Material examined.

Daya Fifi, 1♂, 23.XI.2012; Tributary of Oued El Fondak, 2♂♂, 10.II.2013, 1♂, 10.IV.2013; Barrage Ajras, 2♂♂, 10.II.2013; Daya Mghara, 6♂♂, 14.II.2013; Oued Zarka, 1♂, 23.II.2015; Oued Tizekhte, 1♂, 2♀♀, 28.II.2015 (sweep net).

##### Distribution.

Italy, Portugal, Spain, First records from Morocco and North Africa.

#### Cheilotrichia (Empeda) minima

Taxon classificationAnimaliaDipteraLimoniidae

(Strobl, 1898)

##### Material examined.

Oued Zendoula, 2♂♂, 31.V.2013; Oued Jnane Niche, 1♂, 1♀, 25.IV.2015 (sweep net).

##### Distribution.

Known from Europe (except for the north) and the south of the Palaearctic as far east as Kyrgyzstan. First records from Morocco and North Africa.

#### Ellipteroides (Ellipteroides) lateralis

Taxon classificationAnimaliaDipteraLimoniidae

(Macquart, 1835)

##### Material examined.

Daya Afrate, 3♂♂, 18.IV.2015 (sweep net).

##### Distribution.

Southern part of Europe, Morocco, Lebanon, Israel and Turkey. First record from the Rif.

#### Erioconopa diuturna

Taxon classificationAnimaliaDipteraLimoniidae

(Walker, 1848)

##### Material examined.

Daya Jbel Zemzem, 1♂, 23.IV.2014; Oued Mezine, 4♂♂, 18.IV.2015; Aïn Bab Tariouente, 2♂♂, 2♀♀, 28.IV.2015 (sweep net).

##### Distribution.

Europe, Morocco, Turkey. First record from the Rif.

#### Erioptera (Erioptera) fuscipennis

Taxon classificationAnimaliaDipteraLimoniidae

Meigen, 1818

##### Material examined.

Aïn El Ma Bared, 2♂♂, 30.III.2012; Oued Amsemlil (Site 1), 1♂, 1♀, 27.IV.2012; Oued Majjou (Pont), 2♂♂, 15.V.2012; Oued Tkaraâ, 1♂, 30.V.2013; Daya near Aïn Afersiw, 3♂♂, 1♀, 11.VI.2013; Aïn Afersiw, 3♂♂, 1♀, 11.VI.2013; Oued Abou Bnar, 2♂♂, 18.V. 2014; Oued Majjou (Zaouiet El habtiyne), 9♂♂, 6♀♀, 12.VII.2014; Daya Amsemlil, 2♂♂, 2.XI.2014; Daya Aïn Jdioui, 12♂♂, 5♀♀, 28.III.2015; Daya Afrate, 2♂♂, 2♀♀, 18.IV.2015; Oued Mezine, 1♂, 18.IV.2015 (sweep net).

##### Distribution.

Europe, Morocco, Algeria, Azerbaijan, Iran.

#### Erioptera (Erioptera) lutea lutea

Taxon classificationAnimaliaDipteraLimoniidae

Meigen, 1804

##### Material examined.

Aïn Boughaba, 1♂, 24.V.2013 (sweep net).

##### Distribution.

Europe, Transcaucasia, Turkey, Israel, ?Iran, Russia, Kazakhstan, Uzbekistan, Tajikistan, Kyrgyzstan. First records from Morocco and North Africa.

#### Gonomyia (Gonomyia) abscondita

Taxon classificationAnimaliaDipteraLimoniidae

Lackschewitz, 1935

##### Material examined.

Maison forestière, 3♂♂, 21.IV.2015 (sweep net).

##### Distribution.

Austria, Bulgaria, Czech Rep., Finland, Germany, Great Britain, Italy, Netherlands, Romania, Slovakia, Sweden, Ukraine. First records from Morocco and North Africa.

#### Gonomyia (Gonomyia) subtenella

Taxon classificationAnimaliaDipteraLimoniidae

(Meigen, 1818)

##### Material examined.

Oued Beni Ouachekradi, 2♂♂, 30.XII.2014, 1♀, 8.I.2015 (reared); Oued Jnane Niche, 1♂, 25.IV.2015; Oued Sidi Yahya Aârab, 1♂, 3♀♀, 25.IV.2015 (sweep net).

##### Distribution.

Czech Rep., Lithuania, Macedonia, Slovakia, Sweden, Morocco (High Atlas), Georgia, Azerbaijan, Iran. First records from Rif and Beni Snassen.

#### Gonomyia (Gonomyia) sicula

Taxon classificationAnimaliaDipteraLimoniidae

Lackschewitz, 1940

##### Material examined.

Oued El Kebir, 1♂, 17.IV.2013; Daya Jbel Zemzem, 7♂♂, 23.IV.2014; Aïn Sidi Brahim Ben Arrif (Site 2), 1♂, 25.IV.2014; Daya Tazia, 1♂, 25.IV.2014; Aïn El Maounzil, 1♂, 21.IV.2015 (sweep net).

##### Distribution.

Italy, Spain, Tunisia, Turkey. First records from Morocco.

#### Hoplolabis (Parilisia) sororcula

Taxon classificationAnimaliaDipteraLimoniidae

(Lackschewitz, 1940)

##### Material examined.

Barrage Ajras, 3♂♂, 1♀, 10.II.2013; Oued Ouringa, 1♂, 19.IV.2013; Oued El Kanar, 1♂, 14.VI.2013; Oued Majjou (Hafa meqlouba), 1♂, 10.V.2014 (sweep net).

##### Distribution.

France, Germany, Italy (incl. Sicily), Portugal, Romania, Slovakia, Spain, Switzerland, Ukraine, Morocco. First records from the Rif.

#### Idiocera (Idiocera) ampullifera

Taxon classificationAnimaliaDipteraLimoniidae

(Stary, 1979)

##### Material examined.

Oued Zag, 1♂, 1♀, 22.V.2015 (sweep net).

##### Distribution.

Egypt, Israel. First record from Morocco.

#### Idiocera (Idiocera) pulchripennis

Taxon classificationAnimaliaDipteraLimoniidae

(Loew, 1856)

##### Material examined.

Oued Jnane Niche, 1♂, 2♀♀, 19.IV.2013, 3♂♂, 1♀, 14.VI.2013; Oued Aârkoub, 1♂, 19.IV.2013, 1♂, 16.III.2014; Aïn Jdioui, 4♂♂, 2♀♀, 28.III.2015 (sweep net).

##### Distribution.

Southern part of Europe, Canary Islands, Morocco, Algeria, Egypt, Transcaucasia, Turkey, Cyprus, Israel, Iran, Central Asia. First record from the Rif.

#### Idiocera (Idiocera) sziladyi

Taxon classificationAnimaliaDipteraLimoniidae

(Lackschewitz, 1940)

##### Material examined.

Oued Zarka, 1♂, 22.V.2014 (light trap).

##### Distribution.

Europe (except for northern countries), Algeria, Egypt, Yemen. First record from Morocco.

#### Ilisia maculata

Taxon classificationAnimaliaDipteraLimoniidae

(Meigen, 1804)

##### Material examined.

Oued Tafoughalt, 2♂♂, 25.XI.2014; Oued Majjou (Hafa meqlouba), 1♂, 27.IV.2015 (sweep net).

##### Distribution.

Europe, Transcaucasia, Turkey , Iran. First records from Morocco and North Africa.

#### Molophilus (Molophilus) obscurus

Taxon classificationAnimaliaDipteraLimoniidae

(Meigen, 1818)

##### Material examined.

Tributary of Oued Ouara, 1♂, 27.IV.2013, 5♂♂, 6.V.2014; Tributary of Oued Majjou, 2♂♂, 03.V.2013; Daya El Ânassar, 1♂, 24.V.2013; Daya Jbel Zemzem, 2♂♂, 23.IV.2014; Aïn El Ma Bared, 1♂, 1♀, 6.V.2014; Daya Rmali, 1♂, 17.V.2014; Oued Amsemlil (Site 1), 1♂, 2.XI.2014; Aïn Mâaze, 1♂, 1.XI.2014; Daya Afrate, 9♂♂, 18.IV.2015; Daya Mtahen, 1♂, 25.V.2015 (sweep net).

##### Distribution.

Europe, Morocco (High Atlas), Transcaucasia, Turkey, Cyprus, Lebanon, Israel. First records from the Rif.

#### Molophilus (Molophilus) propinquuspropinquus

Taxon classificationAnimaliaDipteraLimoniidae

(Egger, 1863)

##### Material examined.

Oued Ouara, 1♂, 7.I.2013 (reared); Oued Farda, 3♂♂, 24.IV.2013; Tributary of Oued Taïda, 1♂, 25.IV.2014; Oued Majjou (Hafa meqlouba), 1♂, 27.IV.2015 (sweep net).

##### Distribution.

Europe, Morocco (High Atlas), Georgia, Turkey. First records from the Rif.

#### Molophilus (Molophilus) testaceus

Taxon classificationAnimaliaDipteraLimoniidae

Lackschewitz, 1940

##### Material examined.

Daya Amsemlil, 1♂, 23.V.2012, 1♂, 1♀, 30.V.2013; Daya Mtahen, 2♀♀, 23.V.2012; Marje El kheyl, 3♂♂, 5♀♀, 30.V.2013; Oued Tkaraâ, 1♂, 30.V.2013; Daya near Aïn Afersiw, 1♂, 11.VI.2013; Daya Mezine, 1♂, 11.VI.2013; Daya Tazia, 1♂, 25.IV.2014; Daya Rmali, 1♂, 17.V.2014 (sweep net). Daya Amsemlil, 1♂, 4♀♀, 9.VII.2013; Daya near Aïn Afersiw, 4♂♂, 1♀, 2.VII.2013 (reared).

##### Distribution.

Only known from Portugal and Spain. First records from Morocco and North Africa.

#### Symplecta (Symplecta) hybrida

Taxon classificationAnimaliaDipteraLimoniidae

(Meigen, 1804)

##### Material examined.

Barrage Ajras, 1♂, 10.II.2013; Barrage Allal El Fassi, 1♂, 15.IV.2014; Oued El Kanar, 1♀, 14.VI.2013, 1♂, 25.IV.2015; Oued Aârkoub, 1♂, 16.III.2014; Oued Jnane Niche, 1♀, 25.IV.2015 (sweep net).

##### Distribution.

Nearctic (Canada, USA, Greenland); widespread in Palaearctic, including North Africa, Central Asia, Mongolia, as far east as North Korea and Japan; Oriental (India, Nepal, Pakistan). First records from the Rif and the Middle Atlas.

#### Symplecta (Trimicra) pilipes

Taxon classificationAnimaliaDipteraLimoniidae

(Fabricius, 1787)

##### Material examined.

Tributary of Oued Hachef, 2♂♂, 2♀♀, 14.II.2013; Oued Aârkoub, 1♂, 19.IV.2013; Oued El Kanar, 2♂♂, 2♀♀, 14.VI.2013; Oued Mezine, 1♂, 18.IV.2015 (sweep net); Aïn Chouk, 1♂, 3.II.2015 (reared).

##### Distribution.

The only cosmopolitan cranefly, widespread in all zoogeographical regions. First records from the Rif.

#### Tasiocera (Dasymolophilus) murina

Taxon classificationAnimaliaDipteraLimoniidae

(Meigen, 1818)

##### Material examined.

Oued Farda, 1♂, 28.III.2013; Oued Amsemlil (Site 3), 4♂♂, 30.V.2013 (sweep net).

##### Distribution.

Europe, Transcaucasia, Turkey. First records from Morocco and North Africa.

### Limoniidae: Limnophilinae

#### Austrolimnophila (Austrolimnophila) latistyla

Taxon classificationAnimaliaDipteraLimoniidae

Stary, 1977

##### Material examined.

Oued Majjou (Hafa meqlouba), 1♂, 27.VI.2015 (sweep net).

##### Distribution.

Croatia, France, Greece, Italy, Portugal, Spain. First record from Morocco and North Africa.

#### Dicranophragma (Brachylimnophila) nemorale

Taxon classificationAnimaliaDipteraLimoniidae

(Meigen, 1818)

##### Material examined.

Aïn Sidi Brahim Ben Arrif (Site 1), 1♂, 25.IV.2014; Tributary Oued Ouara, 2♀♀, 27.IV.2013; Oued Majjou (Hafa meqlouba), 1♂, 10.V.2014 (sweep net).

##### Distribution.

Widespread in the Palaearctic. First records from the Rif.

#### Euphylidorea crocotula

Taxon classificationAnimaliaDipteraLimoniidae

(Seguy, 1941)

##### Material examined.

Aïn Bab Tariouente, 3♂♂, 28.IV.2015 (sweep net).

##### Distribution.

Spain (Granada), Morocco (High Atlas). First record from the Rif.

#### Euphylidorea (Euphylidorea) dispar

Taxon classificationAnimaliaDipteraLimoniidae

(Meigen, 1818)

##### Material examined.

Oued at 15 Km from Fifi, 1♀, 26.III.2014 (sweep net).

##### Distribution.

Widespread in Europe, recorded from Russia. First records from Morocco and North Africa.

#### Hexatoma (Hexatoma) bicolor

Taxon classificationAnimaliaDipteraLimoniidae

(Meigen, 1818)

##### Material examined.

Oued at 15 Km from Fifi, 1♂, 27.IV.2013, 1♀, 26.III.2014, 3♂♂, 6.V.2014; Tributary of Oued Ouara, 1♀, 27.IV.2013; Oued Madissouka, 1♂, 18.V.2014; Oued Majjou (Hafa meqlouba), 1♀, 10.V.2014; Aïn Quanquben, 1♂, 28.IV.2015; Oued Tkaraâ, 2♂♂, 7.V.2015; Oued Tamerte, 1♂, 1♀, 7.V.2015 (sweep net).

##### Distribution.

Europe, Georgia, Turkey. First records from Morocco and North Africa.

#### Pseudolimnophila (Pseudolimnophila) sepium

Taxon classificationAnimaliaDipteraLimoniidae

(Verrall, 1886)

##### Material examined.

Daya Tazia, 11♂♂, 8♀♀, 23.IV.2012; Aïn Sidi Brahim Ben Arrif (2), 2♂♂, 2♀♀, 17.IV.2013; Aïn Afersiw, 1♀, 11.VI.2013; Oued Majjou (Hafa meqlouba), 1♂, 10.V.2014 (sweep net). Daya Tazia, 1♂, 2♀♀, 14.V.2013; Oued Taïda, 1♂, 12.V.2014; Aïn Sidi Brahim Ben Arrif (Site 2), 2♂♂, 1♀, 26.V.2014 (reared).

##### Distribution.

Europe, Morocco (Rif), Transcaucasia, Turkey, Turkmenistan, Kyrgyzstan, Afghanistan.

### Limoniidae: Limoniinae

#### Dicranomyia (Dicranomyia) affinis

Taxon classificationAnimaliaDipteraLimoniidae

(Schummel, 1829)

##### Material examined.

Oued Amsemlil (Site 1), 1♂, 27.IV.2012; Daya Mtahen, 10♂♂, 1♀, 27.IV.2012; Oued Tkaraâ, 1♂, 27.IV.2012; Marj El Kheyl, 2♀♀, 30.V.2013; Aïn Sidi Brahim Ben Arrif (Site 2), 5♂♂, 1♀, 17.IV.2013, 3♂♂, 1♀, 25.IV.2014; Aïn Sidi Brahim Ben Arrif (Site 1), 10♂♂, 5♀♀, 25.IV.2014; Daya Tazia, 14♂♂, 4♀♀, 25.IV.2014; Aïn El Maounzil, 6♂♂, 4♀♀, 21.IV.2015; Aïn El Malâab, 1♂, 1♀, 21.IV.2015; Daya near Aïn Afersiw, 5♂♂, 3♀♀, 18.IV.2015; Daya Mtahen, 4♀♀, 7.V.2015; Aïn Bab Tariouente, 4♂♂, 28.IV.2015 (sweep net).

##### Distribution.

Great Britain, Ireland, Poland, Morocco (High Atlas).

#### Dicranomyia (Dicranomyia) chorea

Taxon classificationAnimaliaDipteraLimoniidae

(Meigen, 1818)

##### Material examined.

Oued Sidi Fares, 1♂, 7.V.2011; Oued Farda, 2♂♂, 2♀♀, 13.II.2013, 7♂♂, 4♀♀, 23.IV.2013; Oued Kelâa, 3♂♂, 13.II.2013; Aïn Ras El Ma, 1♂, 3.V.2013; Oued Jnane Niche, 2♂♂, 2♀♀, 25.IV.2015; Cascade Chrafate, 1♂, 2♀♀, 28.IV.2015; Maison forestière, 2♀♀, 21.IV.2015; Oued El Kanar, 1♂, 25.IV.2015 (sweep net). Tributary of Oued Zarka, 4♂♂, 1♀, 9.XII.2013 (reared).

##### Distribution.

Nearctic (Canada), Europe, Morocco, Transcaucasia, Turkey, Cyprus, Israel, Iran. First records from the Rif.

#### Dicranomyia (Dicranomyia) goritiensis

Taxon classificationAnimaliaDipteraLimoniidae

(Mik, 1864)

##### Material examined.

Oued Majjou (Hafa meqlouba), 1♂, 3.V.2013, 2♂♂, 10.V.2014; Cascade Chrafate, 1 ♀, 24.V.2013; Oued El Koub, 1♂, 31.V.2013; Cascade Zarka, 1♂, 14.XI.2013; Âounsar Aheramen, 1♂, 2♀♀, 10.V.2014, 2♂, 27.IV.2015; Maison forestière, 1♂, 21.IV.2015 (sweep net).

##### Distribution.

Europe, Morocco, Algeria, Georgia, Turkey, Israel. First records from the Rif.

#### Dicranomyia (Dicranomyia) longicollis

Taxon classificationAnimaliaDipteraLimoniidae

(Macquart, 1846)

##### Material examined.

Oued Aârate, 4♂♂, 3♀♀, 26.III.2014; Barrage Moulay Bouchta, 2♂♂, 5.IV.2014; Barrage Allal El Fassi, 1♂, 15.IV.2014; Oued El Kebir, 1♀, 23.IV.2014 (sweep net).

##### Distribution.

Portugal, Spain, Morocco, Algeria. First records from the Rif and the Middle Atlas.

#### Dicranomyia (Dicranomyia) modesta

Taxon classificationAnimaliaDipteraLimoniidae

(Meigen, 1818)

##### Material examined.

Oued Farda, 1♂, 24.IV.2013; Oued El Kanar, 4♂♂, 3♀♀, 14.VI.2013, 5♂♂, 10♀♀, 25.IV.2015; Oued Jnane Niche, 2♂♂, 16.III.2014; Oued Sidi Yahya Aârab, 4♂♂, 1♀, 25.IV.2015 (sweep net).

##### Distribution.

Nearctic (Canada, USA, Greenland), widespread over Palaearctic, including Central Asia and Mongolia, as far east as Far East of Russia and Japan. First records from Morocco and North Africa.

#### Dicranomyia (Dicranomyia) novemmaculata

Taxon classificationAnimaliaDipteraLimoniidae

(Strobl, 1906)

##### Material examined.

Tributary of Oued el Fondak, 1♂, 10.II.2013; Oued Aârate, 9♂♂, 4♀♀, 26.III.2014 (sweep net).

##### Distribution.

Gibraltar, Portugal, Spain, Algeria. First records from Morocco.

#### Dicranomyia (Melanolimonia) hamata

Taxon classificationAnimaliaDipteraLimoniidae

Becker, 1908

##### Material examined.

Tributary of Oued El Kebir, 2♂♂, 1♀, 18.III.2011; Oued Aârate, 2♂♂, 26.III.2014; Aïn Sidi Brahim Ben Arrif (Site 1), 2♂♂, 1♀ 25.IV.2014; Daya Tazia, 1♂, 25.IV.2014 (sweep net).

##### Distribution.

France, Portugal, Spain, Turkey. First records from Morocco and North Africa.

#### Dicranomyia (Dicranomyia) ventralis

Taxon classificationAnimaliaDipteraLimoniidae

(Schummel, 1829)

##### Material examined.

Lake Badriouen, 2♂♂, 26.IV.2011 (sweep net).

##### Distribution.

Europe, Egypt, Azerbaijan, Turkey, Israel, Iran, Russia, Kazakhstan, Kyrgyzstan, Afghanistan, North Korea, India. First records from Morocco and North Africa.

#### Dicranomyia (Melanolimonia) morio

Taxon classificationAnimaliaDipteraLimoniidae

(Fabricius, 1787)

##### Material examined.

Seguia Mtahen, 1♂, 30.V.2013; Daya near Aïn Afersiw, 2♀♀, 11.VI.2013; Daya Jbel Zemzem, 1♂, 23.IV.2014 (sweep net).

##### Distribution.

Europe, Morocco, Transcaucasia, Turkey, Iran, Mongolia. First records from the Rif.

#### Dicranoptycha fuscescens

Taxon classificationAnimaliaDipteraLimoniidae

(Schummel, 1829)

##### Material examined.

Oued Mlilah, 3♀♀, 31.V.2013; Oued Loukous, 4♂♂, 1♀, 6.V.2015; Oued Zendoula, 1♀, 6.V.2015 (sweep net).

##### Distribution.

Europe, Morocco, Algeria, Transcaucasia, Turkey, Cyprus, Lebanon, Israel, ?Kazakhstan, Mongolia.

#### Helius (Helius) hispanicus

Taxon classificationAnimaliaDipteraLimoniidae

Lackschewitz, 1928

##### Material examined.

Oued Amsemlil (Site 3), 1♀, 30.V.2013 (sweep net).

##### Distribution.

Great Britain, Spain, Portugal, Morocco (High Atlas), Georgia, Azerbaijan, Turkey, Cyprus, Iran. First record from the Rif.

#### Helius (Helius) pallirostris

Taxon classificationAnimaliaDipteraLimoniidae

Edwards, 1921

##### Material examined.

Aïn Chouk, 1♂, 1♀, 25.II.2015 (reared).

##### Distribution.

Europe, Tunisia, Azerbaijan, Israel, Iran, Central Asia. First record from Morocco.

#### Limonia nubeculosa

Taxon classificationAnimaliaDipteraLimoniidae

(Meigen, 1804)

##### Material examined.

Tributary of Oued El Fondak, 1♀, 10.II.2013; Aïn Ras El Ma, 1♀, 3.V.2013; Aïn Boughaba, 3♂♂, 4♀♀, 24.V.2013; Oued Azila, 1♀, 27.VI.2013; Âounsar Aheramen, 2♂♂, 1♀, 10.V.2014; Aïn Takhninjoute, 1♂, 17.V.2014; Maison forestière, 1♂, 4♀♀, 17.V.2014; Oued Madissouka, 1 ♂, 18.V.2014; Aïn Quanquben, 1♀, 28.IV.2015 (sweep net).

##### Distribution.

Nearctic (Canada, USA), widespread over Palaearctic, including North Africa, Central Asia, and Mongolia, as far east as Far East of Russia, North Korea, and Japan. Recorded from Morocco in the High Atlas. First records from the Rif.

#### Limonia phragmitidis

Taxon classificationAnimaliaDipteraLimoniidae

(Schrank, 1781)

##### Material examined.

Aïn Quanquben, 1 ♂, 27.VI.2013 (sweep net).

##### Distribution.

Widespread in the West Palaearctic, also known from Russia, Kazakhstan and Kyrgyzstan. Recorded from Morocco (Middle Atlas). First record from the Rif.

### Pediciidae

#### Dicranota (Paradicranota) landrocki

Taxon classificationAnimaliaDipteraPediciidae

Czizek, 1931

##### Material examined.

Oued Ouara, 2♂♂, 22.XI.2013; Assif Haouz, 1♂, 17.IV.2014; Oued Taïda, 1♀, 25.IV.2014; Aïn Sidi Brahim Ben Arrif (Site 2), 2♂♂, 25.IV.2014; Âounsar Aheramen, 1♂, 10.V.2014, 1♂, 27.IV.2015; Oued Tizekhte, 1♂, 28.II.2015; Oued Mezine, 1♂, 18.IV.2015; Maison forestière, 32♂♂, 2♀♀, 21.IV.2015; Aïn Bab Tariouente, 1♂, 28.IV.2015 (sweep net).

##### Distribution.

Europe, Russia (North Caucasus), Morocco (Rif), Transcaucasia Lebanon, Tajikistan. First record from the High Atlas.

#### Dicranota (Ludicia) claripennis

Taxon classificationAnimaliaDipteraPediciidae

(Verrall, 1888)

##### Material examined.

Oued Amsemlil (Site 1), 1♂, 16.XII.2013; Maison forestière, 1♂, 21.IV.2015 (sweep net).

##### Distribution.

Austria, Belgium, France, Germany, Great Britain, Ireland, Italy, Netherlands, Spain. First records from Morocco and North Africa.

#### Tricyphona (Tricyphona) immaculata

Taxon classificationAnimaliaDipteraPediciidae

(Meigen, 1804)

##### Material examined.

Maison forestière, 1♂, 21.IV.2015; Daya Mtahen, 1♂, 7.V.2015 (sweep net).

##### Distribution.

Europe, Transcaucasia, Turkey, Lebanon, West Siberia, Central Asia. First records from Morocco and North Africa.

## Discussion

Altogether a number of 570 specimens, most of which (521) belongs to the Limoniidae distributed in 20 genera, whereas only 49 specimens belong to the Pediciidae distributed in 2 genera.

This study provides an important contribution to the short-palped crane flies fauna of North Africa and Morocco, particularly to the Rif region where 35 species were recorded from the the first time. The most abundant species was Dicranomyia (Dicranomyia) affinis, ﻿with 86 specimens, followed successively by, Erioptera (Erioptera) fuscipennis (56), Dicranota (Paradicranota) landrocki (45), Dicranomyia (Dicranomyia) chorea (35), Pseudolimnophila (Pseudolimnophila) sepium (32), Dicranomyia (Dicranomyia) modesta (30) and Molophilus (Molophilus) testaceus (29). The most frequently collected species were Erioptera (Erioptera) fuscipennis (12 sites), Dicranomyia (Dicranomyia) affinis (11 sites), Molophilus (Molophilus) obscurus (10 sites), followed by Dicranomyia (Dicranomyia) chorea,﻿ *Limonia nubeculosa* and Dicranota (Paradicranota) landrocki, all three collected in 9 sites.

18 species of Limoniidae and 2 species of Pediciidae are newly recorded from Morocco, of which 15 species are provided to be new to North Africa. Those new records extend the distribution of the species to most southern localities of the western Palearctics in Africa. Idiocera (Idiocera) ampullifera represents the most southern record (Sahara) of the Limoniidae in Morocco.

Including our results, the total number of species of Limoniidae and Pediciidae known from Morocco now increases from 53 species to 73 (see chekclist).

These new data of crane flies fauna reflect the variety of suitable habitats prospected, such as are springs, mountain rivers and streams, and stagnant water mainly belonging to forest areas. This suggests that many more species and subspecies can yet be found in Morocco, that enjoys a wide variety of geographical and climatic properties.

### Check-list of the Moroccan short-palped crane flies

Limoniidae

**Chioneinae**

*Baeoura ebenina* Starý, 1981

*Baeoura staryi* Driauach & Belqat, 2015

Cheilotrichia (Empeda) cinerascens (Meigen, 1804)

Cheilotrichia (Empeda) fuscohalterata (Strobl, 1906)*

Cheilotrichia (Empeda) minima (Strobl, 1898)*

Ellipteroides (Ellipteroides) lateralis (Macquart, 1835)

Ellipteroides (Protogonomyia) alboscutellatus (Von Roser, 1840)

Ellipteroides (Protogonomyia) hutsoni (Stary, 1971)

*Erioconopa diuturna* (Walker, 1848)

*Erioconopa symplectoides* (Kuntze, 1914)

Erioptera (Erioptera) fuscipennis Meigen, 1818

Erioptera (Erioptera) lutea lutea Meigen, 1804*

Erioptera (Erioptera) transmarina Bergroth, 1889

Gonomyia (Gonomyia) abscondita Lackschewitz, 1935*

Gonomyia (Gonomyia) subtenella Savchenko, 1972

Gonomyia (Gonomyia) sicula Lackschewitz, 1940*

Gonomyia (Gonomyia) tenella (Meigen, 1818)

Hoplolabis (Parilisia) obtusiapex (Savchenko, 1982)

Hoplolabis (Parilisia) punctigera (Lackschewitz, 1940)

Hoplolabis (Parilisia) sororcula (Lackschewitz, 1940)

Idiocera (Euptilostena) jucunda (Loew, 1873)

Idiocera (Idiocera) ampullifera (Stary, 1979)*

Idiocera (Idiocera) pulchripennis (Loew, 1856)

Idiocera (Idiocera) sziladyi (Lackschewitz, 1940)*

*Ilisia maculata* (Meigen, 1804)*

Molophilus (Molophilus) ibericus Stary, 2011

Molophilus (Molophilus) obscurus (Meigen, 1818)

Molophilus (Molophilus) propinquus propinquus (Egger, 1863)

Molophilus (Molophilus) testaceus Lackschewitz, 1940*

Symplecta (Symplecta) hybrida (Meigen, 1804)

Symplecta (Trimicra) pilipes (Fabricius, 1787)

Tasiocera (Dasymolophilus) murina (Meigen, 1818)*

Dactylolabinae

Dactylolabis (Dactylolabis) symplectoidea Egger, 1863

Limnophilinae

Austrolimnophila (Austrolimnophila) latistyla Stary, 1977*

Dicranophragma (Brachylimnophila) adjunctum (Walker, 1848)

Dicranophragma (Brachylimnophila) nemorale (Meigen, 1818)

*Eloeophila maroccana* Stary, 2009

Euphylidorea (Euphylidorea) crocotula (Seguy, 1941)

Euphylidorea (Euphylidorea) dispar (Meigen, 1818)*

Euphylidorea (Euphylidorea) lineola (Meigen, 1804)

Hexatoma (Hexatoma) bicolor (Meigen, 1818)*

Hexatoma (Hexatoma) gaedii (Meigen, 1830)

Pseudolimnophila (Pseudolimnophila) sepium (Verrall, 1886)

Limoniinae

Dicranomyia (Dicranomyia) affinis (Schummel, 1829)

Dicranomyia (Dicranomyia) chorea (Meigen, 1818)

Dicranomyia (Dicranomyia) didyma (Meigen, 1804)

Dicranomyia (Dicranomyia) goritiensis (Mik, 1864)

Dicranomyia (Dicranomyia) longicollis (Macquart, 1846)

Dicranomyia (Dicranomyia) lutea Meigen

Dicranomyia (Dicranomyia) mitis (Meigen, 1830)

Dicranomyia (Dicranomyia) modesta (Meigen, 1818)*

Dicranomyia (Dicranomyia) novemmaculata (Strobl, 1906)*

Dicranomyia (Dicranomyia) ventralis (Schummel, 1829)*

Dicranomyia (Glochina) sericata (Meigen, 1830)

Dicranomyia (Melanolimonia) hamata Becker, 1908*

Dicranomyia (Melanolimonia) morio (Fabricius, 1787)

*Dicranomyia majuscula* Pierre, 1924

*Dicranoptycha fuscescens* (Schummel, 1829)

*Geranomyia caloptera* (Mik, 1867)

*Geranomyia obscura* Strobl, 1900

Helius (Helius) hispanicus Lackschewitz, 1928

Helius (Helius) pallirostris Edwards, 1921*

*Limonia flavipes* (Fabricius, 1787)

*Limonia hercegovinae* (Strobl, 1898)

*Limonia macrostigma* (Schummel, 1829)

*Limonia nubeculosa* Meigen, 1804

*Limonia phragmitidis* (Schrank, 1781)

Pediciidae

**Pediciinae**

Dicranota (Dicranota) bimaculata (Schummel, 1829)

Dicranota (Dicranota) irregularis Pierre, 1922

Dicranota (Paradicranota) candelisequa Stary, 1981

Dicranota (Paradicranota) landrocki Czizek, 1931

Dicranota (Ludicia) claripennis (Verrall, 1888)*

**Tricyphona (Tricyphona) immaculata (Meigen, 1804)**

## Supplementary Material

XML Treatment for Cheilotrichia (Empeda) cinerascens

XML Treatment for Cheilotrichia (Empeda) fuscohalterata

XML Treatment for Cheilotrichia (Empeda) minima

XML Treatment for Ellipteroides (Ellipteroides) lateralis

XML Treatment for Erioconopa diuturna

XML Treatment for Erioptera (Erioptera) fuscipennis

XML Treatment for Erioptera (Erioptera) lutea lutea

XML Treatment for Gonomyia (Gonomyia) abscondita

XML Treatment for Gonomyia (Gonomyia) subtenella

XML Treatment for Gonomyia (Gonomyia) sicula

XML Treatment for Hoplolabis (Parilisia) sororcula

XML Treatment for Idiocera (Idiocera) ampullifera

XML Treatment for Idiocera (Idiocera) pulchripennis

XML Treatment for Idiocera (Idiocera) sziladyi

XML Treatment for Ilisia maculata

XML Treatment for Molophilus (Molophilus) obscurus

XML Treatment for Molophilus (Molophilus) propinquuspropinquus

XML Treatment for Molophilus (Molophilus) testaceus

XML Treatment for Symplecta (Symplecta) hybrida

XML Treatment for Symplecta (Trimicra) pilipes

XML Treatment for Tasiocera (Dasymolophilus) murina

XML Treatment for Austrolimnophila (Austrolimnophila) latistyla

XML Treatment for Dicranophragma (Brachylimnophila) nemorale

XML Treatment for Euphylidorea crocotula

XML Treatment for Euphylidorea (Euphylidorea) dispar

XML Treatment for Hexatoma (Hexatoma) bicolor

XML Treatment for Pseudolimnophila (Pseudolimnophila) sepium

XML Treatment for Dicranomyia (Dicranomyia) affinis

XML Treatment for Dicranomyia (Dicranomyia) chorea

XML Treatment for Dicranomyia (Dicranomyia) goritiensis

XML Treatment for Dicranomyia (Dicranomyia) longicollis

XML Treatment for Dicranomyia (Dicranomyia) modesta

XML Treatment for Dicranomyia (Dicranomyia) novemmaculata

XML Treatment for Dicranomyia (Melanolimonia) hamata

XML Treatment for Dicranomyia (Dicranomyia) ventralis

XML Treatment for Dicranomyia (Melanolimonia) morio

XML Treatment for Dicranoptycha fuscescens

XML Treatment for Helius (Helius) hispanicus

XML Treatment for Helius (Helius) pallirostris

XML Treatment for Limonia nubeculosa

XML Treatment for Limonia phragmitidis

XML Treatment for Dicranota (Paradicranota) landrocki

XML Treatment for Dicranota (Ludicia) claripennis

XML Treatment for Tricyphona (Tricyphona) immaculata

## References

[B1] Driauach O Belqat B (2015) A new species of the genus *Baeoura* from Morocco, with a key to the West Palaearctic species (Diptera: Tipuloidea: Limoniidae). ZooKeys 532: 99–105. doi: 10.3897/zookeys.532.5994 26692808 10.3897/zookeys.532.5994 PMC4668895

[B2] Driauach O Belqat B De Jong H (2013) A first checklist of the short-palped crane flies (Diptera: Limoniidae, Pediciidae) of Morocco. Boletín de la Sociedad Entomológica Aragonesa 53: 187–190.

[B3] Eiroa E (2000) Primera cita de *Dicranoptycha fusescens* (Schummel, 1829) para Marruecos (Diptera: Limoniidae). Boletín de la Asociación Espanola de Entomología 24: 204.

[B4] De Jong H Oosterbroek P Gelhaus J Reusch H Young C (2008) Global biodiversity of craneflies (Insecta, Diptera:Tipulidea or Tipulidae sensu lato) in freshwater. Hydrobiologia 595: 457–467. doi: 10.1007/s10750-007-9131-0

[B5] Oosterbroek P (2015) Catalogue of the Craneflies of the World (Insecta, Diptera, Nematocera, Tipuloidea). http://ccw.naturalis.nl/index.php [version 26 November 2015]

[B6] Pârvu C Rãzvan PM Rãzvan Z (2006) Faunistic data on some dipteran families (Insecta: Diptera) from Morocco. Travaux du Muséum d’Histoire Naturelle “Grigore Antipa” 49: 271–281.

[B7] Pierre C (1922a) Nematocera Polyneura recueillis au Maroc par M. Charles Alluaud (1919–1920) (Insectes Diptères). Bulletin de la Société des Sciences naturelles du Maroc 1: 21–24.

[B8] Pierre C (1922b) Nematocera Polyneura recueillis au Maroc par M. Charles Alluaud (2e liste, (1920–1921) (Insectes Diptères). Bulletin de la Société des Sciences naturelles du Maroc 1: 148–151.

[B9] Pierre C (1924) Nematocera Polyneura receuillis au Maroc par M. Charles Alluaud (3e liste, 1922–1923) (Insectes Diptères). Bulletin de la Société des Sciences naturelles du Maroc 4: 198–201.

[B10] Reusch H Oosterbroek P (1997) Diptera Limoniidae and Pediciidae, Short-palped Crane Flies. In: Nilsson A (Ed.) Aquatic Insects of North Europe 2: 105–132.

[B11] Savchenko EN Oosterbroek P Starý J (1992) Family Limoniidae. Catalogue of Palaearctic Diptera 1: 183–369.

[B12] Séguy E (1941a) Récoltes de R. Paulian et A. Villiers dans le haut Atlas marocain, 1938, (XVIIe note) Diptères. Revue Française d’Entomologie 8: 25–33.

[B13] Séguy E (1941b) Diptères receuillis par M. Berland dans le sud marocain. Annales de la Société Entomologique de France 110: 1–23.

[B14] Starý J (1971) A new palaearctic representative of the subgenus *Protogonomyia* Alexander (Diptera, Tipulidae). Acta Entomologica Bohemoslovaca 68: 319–321.

[B15] Starý J (1974) The identity of Gonomyia (Idiocera) sexguttata (Diptera, Tipulidae). Acta Entomologica Bohemoslovaca 71: 136–140.

[B16] Starý J (2006) Hoplolabis (Parilisia) species related to H. (P.) punctigera (Lackschewitz, 1940) and H. (P.) spinosa (Nielsen, 1953) with the description of a new species (Diptera,Limoniidae). Studia dipterologica 13: 115–125.

[B17] Starý J (2009) West Palaearctic species of the genus *Eloeophila* (Diptera: Limoniidae). European Journal of Entomology 106: 425–440. doi: 10.14411/eje.2009.054

[B18] Starý J (2011) Descriptions and records of the Palaearctic *Molophilus* Curtis (Diptera, Limoniidae). Zootaxa 2999: 45–62.

[B19] Starý J Kubik S Bartak M (2005) Limoniidae. In: Bartak M Kubik S (Eds) Diptera of Podyji National Park and its environs. Ceska Zemedelska Univerzita v Praze, 24–32.

[B20] Starý J Oosterbroek P (2008) New records of West Palaearctic Limoniidae, Pediciidae and Cylindrotomidae (Diptera) from the collections of the Zoological Museum, Amsterdam. Zootaxa 1922: 1–20.

[B21] Vaillant F (1956) Recherches sur la faune madicole (Hygropetriques.l.) de France, de Corse et d’Afrique de Nord. Mémoires du Museum National d’Histoire Naturelle (N.S.), Série A, Zoologie 11: 1–257.

